# Barriers and Enablers to Asynchronous Secure Messaging Use in Hospital-Based Ambulatory Specialty Clinics in Ontario, Canada: Convergent Mixed Methods Study

**DOI:** 10.2196/92432

**Published:** 2026-10-22

**Authors:** Vess Stamenova, Taylor Pratt, Hayden Estabrook, Lori Wasserman, Onil Bhattacharyya, Payal Agarwal, Geetha Mukerji

**Affiliations:** 1 Ted Rogers School of Management, Toronto Metropolitan University, 55 Dundas Street West, 9th Floor, Toronto, Ontario, ON, M5G 2C3, Canada, 1 4162680985; 2 Institute for Health System Solutions and Virtual Care, Women's College Hospital, Toronto, ON, Canada; 3 Decision Support, Women's College Hospital, Toronto, ON, Canada; 4 Psychiatry, Women's College Hospital, Toronto, ON, Canada

**Keywords:** secure messaging, specialist care, telehealth, Canada, electronic health records, virtual care, mixed methods

## Abstract

**Background:**

The COVID-19 pandemic accelerated the adoption of virtual care, including patient-to-provider asynchronous secure messaging (ASM). However, while ASM offers access and convenience, the surge in message volume can increase physician workload and contribute to burnout. Furthermore, it has raised additional concerns about message appropriateness, medicolegal liability, and sustainability of this communication virtual care modality.

**Objective:**

This study aims to explore the experience with ASM in Ontario in specialty ambulatory care to inform current practice support strategies. We sought to characterize messaging usage quantitatively while also exploring experience with messaging, contextual barriers, and facilitators qualitatively.

**Methods:**

We used a convergent mixed methods design, analyzing aggregated electronic health record (EHR) usage and qualitative interview data. The study was conducted at a large ambulatory care hospital in Ontario, Canada, across four high-messaging specialty clinics: mental health, rheumatology, dermatology, and surgery. Quantitative data (October 2019 to Octtober 2022) included message volumes, response patterns, and timing. Semistructured interviews with physicians, nurses, administrative staff, and patients (n=19), conducted in 2022 explored ASM workflows, barriers, and facilitators. Quantitative data were analyzed descriptively, stratified by clinic. Qualitative data were analyzed using thematic analysis, informed by an interpretative phenomenological orientation. Findings from the two strands were then compared to identify areas of convergence and divergence.

**Results:**

Message volumes surged post pandemic, particularly in mental health, ranging from about 6000 in surgery, rheumatology, and dermatology to close to 13,000 messages in mental health across the study period. Message rate per patient per month was 0.6 (SD 0.2; mental health), 0.5 (SD 0.2; rheumatology), 0.2 (SD 0.2; dermatology), and 0.2 (SD 0.1; surgery). Physicians averaged 12.6 (SD 8.0) messages/month (rheumatology) and 7.6 (SD 4.4) messages/month (mental health) in clinics without nursing support. Nurses handled 27.4 (SD 11.6) messages/month (surgery) and 19.2 (SD 14.1) messages/month (dermatology). Three percent (1263/38,370) to 10% (3619/36,022) of provider messages were sent outside regular hours. The majority of conversations (2156/6859, 46% to 6913/13,615, 62%) had only a single reply. Physicians reported challenging experiences due to increased workload, lack of compensation, and poor integration into local clinical workflows. High patient-to-physician ratios and limited nursing support for message triage were associated with a poor messaging experience. Patients and administrative staff valued messaging for its convenience, accessibility, and efficiency. A key finding was insufficient engagement across all user groups in decisions about messaging implementation.

**Conclusions:**

The study highlights a disconnect between the high perceived value of ASM for patients and administrative staff and the challenging experiences of physicians. Successful implementation requires thoughtful integration into care models, clear guidelines for appropriate use, and proper triage and “channel management” to guide patients to appropriate modalities. Future research should explore triaging algorithms as part of a digital front door, specialty-specific variations, and the crucial role of nursing staff in message channel management.

## Introduction

Alongside the dramatic increase in virtual visits (phone and video) during the COVID-19 pandemic [1], there was a significant rise of patient-to-provider asynchronous “secure messages” (ASM) [2,3]. Secure messages are messages sent through a secure web-based platform, such as a patient portal, that are asynchronous, similar to email messages. Patients may use ASM to share clinical updates, seek medical guidance, request prescription renewals, follow up on test results, and manage their appointments [4]. The use of ASM offers a multitude of benefits for patients, clinicians, and administrative staff [5,6]. They are convenient and offer a document record of all health care communications; furthermore, they may substitute for some synchronous visits and can be associated with lower treatment costs with comparable clinical outcomes [7-9]. For providers and administrative staff, secure messaging can be convenient and enables efficient handling of routine tasks such as prescription renewals, appointments, and nonurgent clinical queries.

While there are perceived benefits to ASM for patients and their care teams, the increased volume and new workload for providers are increasingly being recognized [6,7]. There has been a notable recognized rise in clinician burnout, often linked to the widespread adoption of electronic health records (EHRs) and digital tools [8-10], and studies have reported that in-basket work (work spent exchanging messages with patients and other health care providers) contributes mostly to physician burden [7,11]. Responding to messages is generally not well integrated into clinical routine care delivery, leading to additional work outside of clinical hours [12-14]. Physicians also often express concerns about the utility of ASM, including the lack of context, incomplete information in messages, as well as the incorrect use of messaging by patients for more urgent requests [15-20]. The challenges of ASM are further exacerbated by the lack of remuneration for these services across many health care systems [21,22]. The experience in the United States with the use of messaging has been mixed [23], and there is limited use of secure messaging in our publicly funded health care system in Ontario, Canada. The Ministry of Health in Ontario introduced a new pilot program in 2024, which enables some physicians to bill for providing clinical encounters through ASM in an attempt to better understand the value proposition within a publicly funded health care system and to assess if it can be well integrated into existing models of care in primary and specialty care in Ontario. This study will explore previous experience with messaging in Ontario prior to this pilot to understand the experience, barriers, and facilitators to inform current practice support strategies with ASM use. Understanding these baseline barriers and facilitators is critical to the pilot’s success, as billing incentives alone are unlikely to resolve structural issues such as inadequate triage support, unclear role delineation, and poor workflow integration. We sought to characterize messaging usage quantitatively while also exploring experience with messaging and contextual barriers and facilitators qualitatively.

## Methods

### Study Setting

The study was conducted at an ambulatory academic hospital located in Ontario, Canada, with 1165 staff, 350 nurses and allied health professionals, and 586 physicians, providing 330,000 in-person visits and 192,000 virtual care visits in a year. Among its many specialties are cardiology, dermatology, endocrinology, gastroenterology, internal medicine, gynecology, psychiatry, respirology, rheumatology, surgery, and oncology. In all departments, except family practice, the hospital has used Epic EHR since 2015. At the time of this project, about 180,000 patients are active users of the Epic-based patient portal myHealthrecord, while about 20,000 patients log in every month. The portal also allows patients to send messages through two distinct pathways: the “Scheduling Requests” pathway allows them to request or change appointments, while the “Medical Advice Requests” pathway allows them to ask medical questions. We conducted this study at four specialty departments at Women’s College Hospital (WCH) that had high use of messaging: mental health, rheumatology, dermatology, and surgery. The clinics were chosen to represent different models of consultation, like single consultations, semiannual visits, or defined episodes of care over several visits.

In Ontario, Canada, most hospitals are publicly funded, but physicians are not employees of the hospital and instead are paid by billing the Ontario Health Insurance Plan (OHIP), so are effectively considered self-employed. On March 14, 2020, the Ontario Ministry of Health introduced new temporary billing codes that allowed all physicians in Ontario to provide care through video or phone to allow for social distancing measures to take effect. Secure messaging and email were not included as a remunerated modality for providing patient care.

In December 2021, the Department of Medicine at the hospital shut down the patient-initiated medical advice requests option due to concerns expressed by physicians about the large volume and adequacy of messages they were receiving, limiting time to support synchronous care delivery. This affected only two of the participating departments: rheumatology and dermatology. Administrative staff, who were still able to receive messages through the Scheduling Request pathway within these two clinical areas, continued to forward messages to physicians if clinical questions were included in the scheduling requests.

In dermatology and surgery, both patient-initiated medical advice requests and scheduling messages were operational during the study. In these two areas, nurses were accountable for triaging messages. Depending on the nature of the message, the triage nurse would either respond or forward messages to the most responsible physician when a clinical decision required their involvement, and the physician would decide how best to address the patient’s request. Conversely, there was no available workflow to support triaging messages or support physicians in handling message responses in rheumatology. Physicians in rheumatology bore full responsibility for triaging, addressing clinical questions, and subsequent follow-up with administrative staff, providing assistance for nonclinical queries for messages sent to the Medical Advice Request workpool. All messages sent to the scheduling messaging workpool within general rheumatology account were reviewed and screened by administrative staff before being forwarded to physicians.

The mental health department’s messages were first screened by administrative staff. The staff handled administrative requests, and any clinical questions were forwarded to the patient’s physician or allied health professional (if the indicated therapist was allied health), similar to the workflow model in the rheumatology department. The responsible clinician responded to most messages in the mental health department.

### Study Design

The research team brought relevant expertise that shaped this study. The principal investigator (VS) is a health services researcher with expertise in virtual care and clinical informatics, including prior research on patient-provider communication technologies. The research coordinator (TP) had experience working on various digital health projects at the Centre for Digital Health Evaluation. This background provided familiarity with the clinical context and messaging workflows, which facilitated rapport-building with participants but also required conscious attention to avoid confirmation bias when interpreting data about technology implementation challenges.

We used a convergent parallel mixed methods study design using aggregated quantitative EHR usage data and qualitative interview data (Figure 1). Quantitative data were examined to investigate the level of use of secure messaging, while qualitative data explored experiences and barriers and facilitators to messaging use in the four hospital-based, ambulatory specialty clinics. Parallel data collection allowed the capture of real-time feedback, while usage patterns evolved due to the evolving adoption of virtual care during the time period. We collected and analyzed quantitative and qualitative data concurrently but independently, and integrated findings during interpretation to characterize understanding of secure messaging use in four ambulatory specialty clinics.

**Figure 1.**
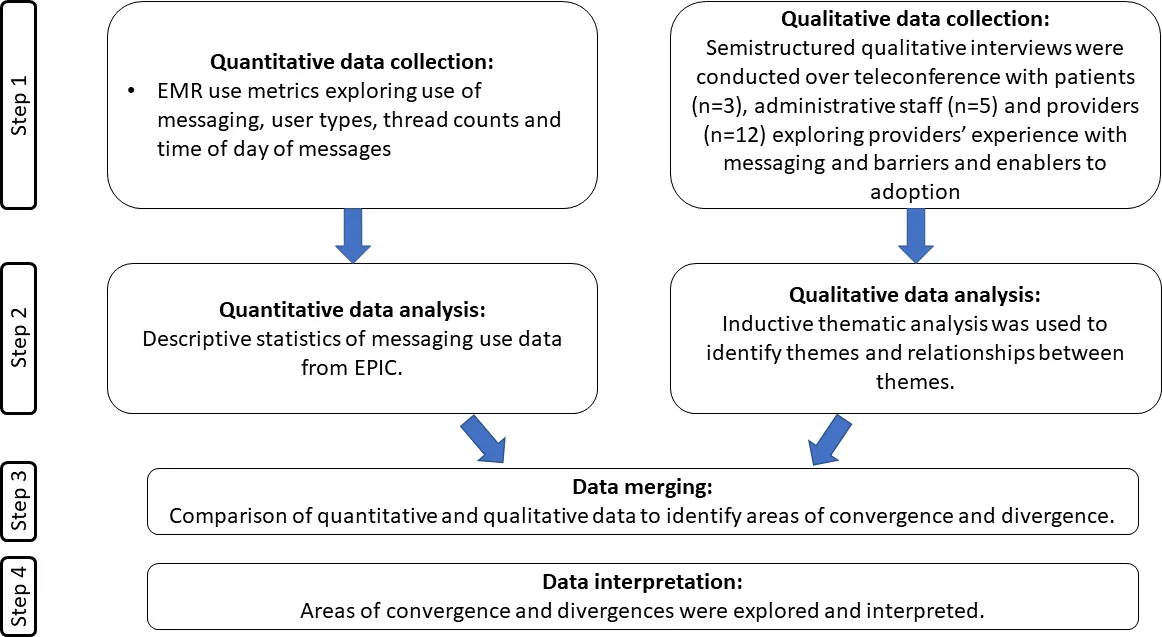
Mixed methods analytical design.

### Ethical Considerations

The study was approved by the Hospital Ethics Assessment Process for Quality Improvement Projects (APQIP # 2022‐0042-P). All participants who participated in the interviews completed an informed consent. Participants were informed that we were conducting a study examining experiences with the use of secure messaging, with the ultimate goal to inform potentially successful models of care that integrate secure messaging. All data were deidentified and stored in a secure location at the hospital.

### Data Collection and Analysis

#### Quantitative EHR Data Collection and Analysis

We conducted a longitudinal time-series study using EHR data from Epic. We examined the number of messages sent by users (patients, physicians, and other clinicians) and volumes of various types of clinic visits (ie, phone, video, and in-person). We classified messages as either “initiated” (first message in a thread) or “response” (second or subsequent message in a thread). Messages were also classified as being sent to the patient or to the staff (from the patient). Data were pulled monthly over 25 months, from 6 months before the onset of the COVID-19 pandemic (October 2019) through October 2022. We collected data from Epic on the department ID, which showed us which department or clinic was data coming from. We also had data on the message category as classified by Epic. The message categories included were patient medical advice requests, information updates, patient scheduling requests; system messages and questionnaire submission messages were excluded from the study. Users were classified according to their Epic user type (eg, physician, nurse, and allied health). Patients and providers were included in the total monthly count of patients and providers when calculations of messages per patient and provider were done. Patients or providers were counted in the denominator if they had at least one appointment per month within their respective clinic. The time a message was sent was also collected. Finally, we also counted the number of messages within a single thread (or conversation). All data were aggregated and processed by the decision support staff at the hospital. Descriptive statistics were used to describe all quantitative data stratified across the four clinics.

#### Qualitative Data Collection and Analysis

The semistructured interviews aimed to understand barriers and facilitators associated with using ASM. We also used the interviews to better understand the pre-existing workflows for messages, as reported by administrative and clinical staff.

Health care professionals (physicians, nurses, and social workers) and administrative staff working at participating clinics were eligible for participation. We used a convenience and snowballing sampling approach by reaching out to key contacts within the participating departments and letting them connect us with other potential participants, including people in their department. Patients who had a visit to the hospital (irrespective of department, modality, or messaging use) within the data collection period (October 2019 to October 2022) were also eligible for participation. Eligible participants (health care professionals, administrative staff, and patients) were required to be 18 years of age or older and proficient in English. We engaged the local hospital patient experience advisors group to support recruitment; advisors distributed information about the study to patients, and interested patients responded directly to the advisors, who then connected them with the study team. Only patients who had an active patient portal account could use ASM. All interview participants provided verbal or written consent to participate in the study. Participants were not compensated for their participation.

Single semistructured interviews of 30‐45 minutes were conducted over Microsoft Teams or the phone with the participant and a single interviewer present. Interviews were conducted by VS (research lead, with 6 years of experience in mixed methods and qualitative research at the time of the study) and TP (research assistant, trained by VS, with approximately one year of qualitative research experience) in 2022, with the participant and a single interviewer present for each interview. They had no prior relationship with the participants. The interviews were audio-recorded and transcribed. Field notes were not taken, and transcripts were not shared with the participants. Health care provider interviews focused on learning about the current messaging models of care, experience with messaging, usage of messaging, and barriers and enablers to adoption of the use of messaging in their clinic. Patient interviews focused on the experience using messaging in their care, and barriers and facilitators of adoption. Questions were tailored to each clinic and participant type. Data saturation was not formally assessed in this study. Due to recruitment challenges, particularly in engaging nursing staff despite multiple attempts, we prioritized obtaining diverse perspectives across the four clinics rather than pursuing saturation within any single stakeholder group.

Audio files were transcribed by either an automatic transcription service through the video conference platform or by a research assistant and were deidentified prior to analysis. Automatically generated transcripts were reviewed against the original audio recordings to verify accuracy prior to analysis. Interviews were analyzed iteratively using thematic analysis to identify common themes and patterns of meaning across interviews [24]. Two coders (VS, PhD and research lead, and TP, BSc and research coordinator; both female) coded the first 3 interviews and met to discuss and develop an initial coding framework. The 2 coders then applied this framework to the remaining interviews using NVivo (Lumivero), meeting regularly to discuss coding decisions and refine their interpretations collaboratively. This iterative discussion supported a more reflexive and nuanced interpretation of the data than either coder could have developed independently. Dependability was addressed through our systematic coding process, where we documented our analytical decisions and maintained consistency in applying codes across transcripts. Themes were discussed among team members at the end, but not with participants. VS has been trained in mixed methods research and had used such methods for 6 years at the time of the study, while TP was trained by VS and had engaged in qualitative data analysis for about a year.

Data analysis was guided by a reflexive thematic analysis approach [24,25], which acknowledges that researchers’ own professional insights and assumptions play an active role in interpreting the meaning of participants’ experiences. Analysis proceeded through familiarization with the transcripts, inductive generation of initial codes, iterative grouping of codes into candidate themes, and review and refinement of themes through discussion among the research team until a coherent set of themes representing patterns of meaning across participants was finalized. We actively sought disconfirming evidence during analysis and triangulated qualitative findings with quantitative messaging data to corroborate or challenge emerging themes. The data were reported using the Consolidated Criteria for Reporting Qualitative Research (COREQ) [26].

### Mixed Methods Data Analysis

To enhance credibility, we used triangulation by integrating multiple data sources (interviews with physicians, nurses, administrative staff, and patients) and combining qualitative and quantitative data to corroborate findings.

After extracting the main qualitative themes and subthemes, we examined the aggregate EHR data to identify points of convergence and divergence between the EHR data and the statements made by the interviewed participants. For example, if providers said that nurses are forwarding all their messages, we looked at EHR data to see the share of messages nurses respond to.

## Results

### Quantitative Results

#### Clinic Characteristics

The participating clinics had different staff representation and patient numbers. Table 1 lists the average number of patients, physicians, nurses, and allied health professionals per month across the clinics, but it does not account for whether physicians were working full-time or part-time at the hospital. Many physicians practice at multiple hospitals.

**Table 1.** Specialty clinics included in the evaluation and their characteristics^a^.

Clinic	Average number of unique patients per month (mean, SD)	Average number of physicians (mean, SD) per month	Average number of nursing staff per month (mean, SD)	Average number of allied health staff per month (mean, SD)	Type of patients
Mental health	1142 (159)	28 (6)	1‐2	12 (2.4)	Chronic, rotations from about 10 weeks to 13 months.
Rheumatology	706 (102)	8-10	0‐1	0	Long-term, chronic patients, with repeat visits over months and years.
Dermatology	1834 (271)	26 (2)	10 (2)	0	Mostly episodic with repeat visits over weeks to a few months.
Surgery	1986 (291)	41 (4)	5 (1)	3 (1)	Acute, episodic care with repeat visits over weeks post-surgery.

a Average numbers are shown as a monthly average. Patients and providers were included in each monthly count if they had at least one appointment per month within their respective clinic, irrespective of whether they used messaging or not.

#### Message Characteristics

Message volumes were highest in the mental health department (12,943 initiated and 12,980 responses each over the October 2019 to October 2022 period), while the other three departments had a total of about 6000 initiated and response messages each (Table 2). The message rate per patient varied, with higher rates in mental health and rheumatology (0.6, SD 0.2 and 0.5, SD 0.2 messages per patient per month in mental health and rheumatology, respectively; details in Table 2 and Figure 2).

**Table 2.** Total volumes of messages and rate of messages per patient across the study period.

Clinic	Initiated, n	Response, n	Initiated patient message rate per month per patient, mean (SD)
Mental health	12,943	12,980	0.6 (0.3)
Rheumatology	6754	6205	0.5 (0.2)
Dermatology	6240	5283	0.2 (0.1)
Surgery	5969	5840	0.2 (0.1)

**Figure 2.**
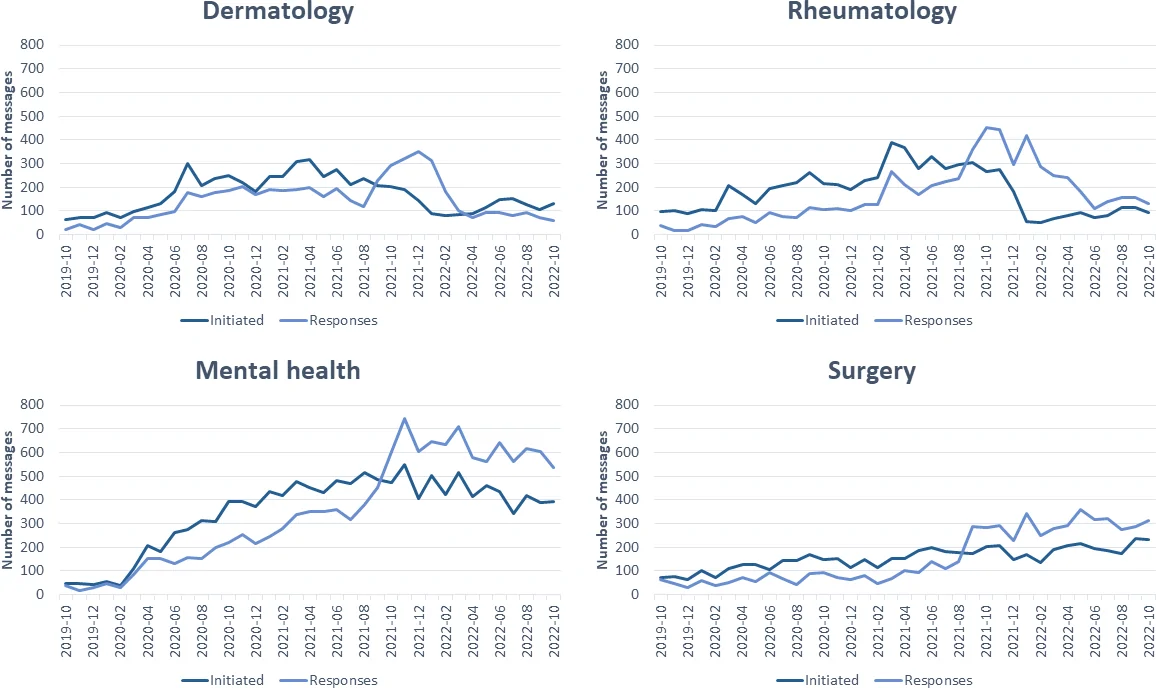
Total number of patient messages.

There was an apparent increase in the number of messages at the onset of the pandemic (March 2020), which was likely due to an increasing number of patients being onboarded on the patient portal to enable video visits. Dermatology and rheumatology switched off the patient-initiated direct-to-physician messaging in December of 2021, and, inherently, the decrease in volumes was seen in these two departments. There was also a trend across all departments in September 2021, with patient responses outnumbering patient-initiated messages. Most departments also saw a substantial increase in provider-initiated messages in July 2021. The reason for these changes was not clear. We observed an uptick in provider-initiated messages in July 2021, and the increased responses in September may represent the continuation of message threads initiated during that period.

In mental health, physicians and allied health professionals were the most frequent clinician-users of messages, initiating and responding to messages in almost equal numbers (Figures 3 and 4). In rheumatology, physicians were the main users of messages, responding to more messages (3469 messages) than they initiated (384 messages over the 37-month study period). Both of these departments had limited or no nursing support. As a result, the average number of messages per physician per month was highest in rheumatology (mean 12.6, SD 7.9) and mental health (mean 7.6, SD 4.3).

**Figure 3.**
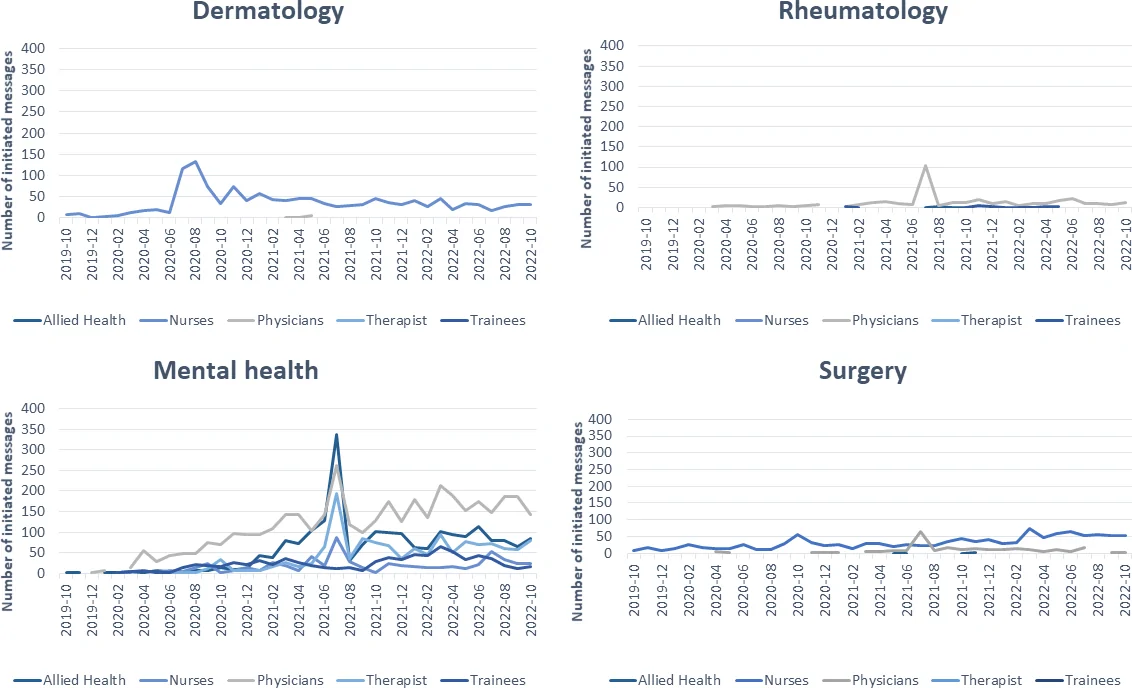
Total number of initiated messages across user types.

Nurses were the primary clinicians responding to messages in surgery (mean 27.3, SD 11.6 messages per month) and in dermatology (mean 5.9, SD 4.0 messages per month).

**Figure 4.**
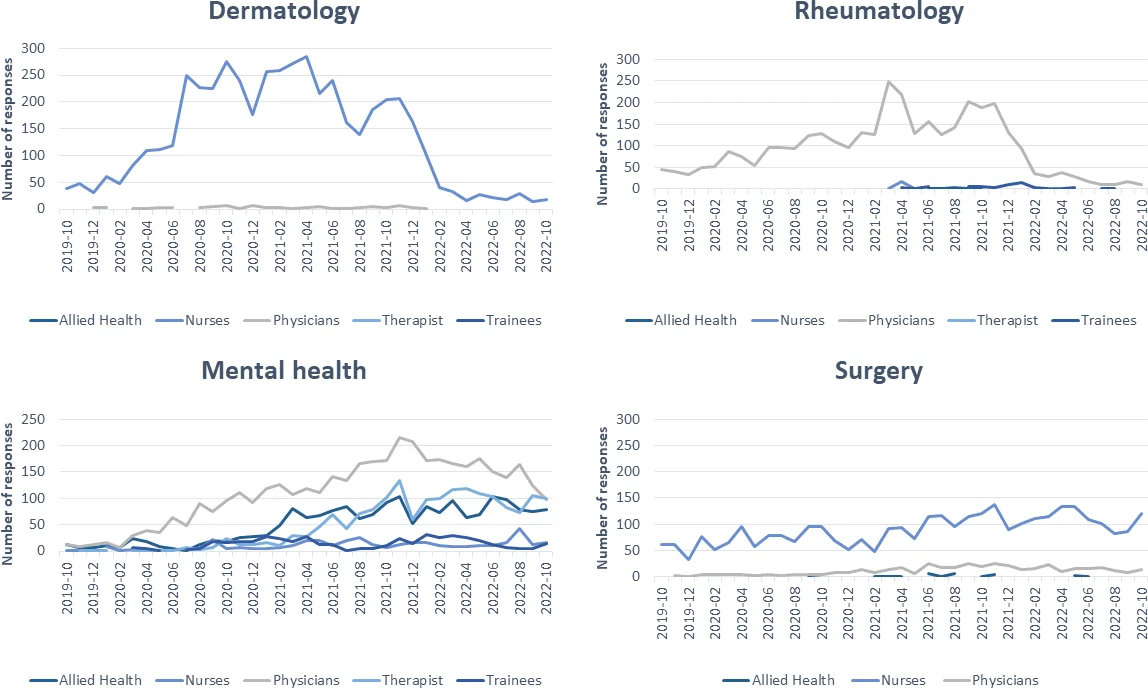
Total number of response messages across user types.

We also examined the number of messages per conversation (Figure 5). A large number of messages were not responded to across all clinics, including both patient-initiated and provider-initiated messages. Across messages sent from patients, 36% (2268/6360) in dermatology, 32% (2221/6859) in rheumatology, 19% (2529/13,615) in mental health, and 43% (1189/2276) in surgery were not responded to by staff. Across messages sent by staff, 38% (1009/2638) in dermatology, 79% (3850/4871) in rheumatology, and 54% (7346/11,225) in mental health and surgery were not responded to by patients. The reasons behind were not clear, as we did not have access to content. It is possible that some of these messages were reminders, or automated messages that were sent by users. Some messages may also have been responded to through telephone.

**Figure 5.**
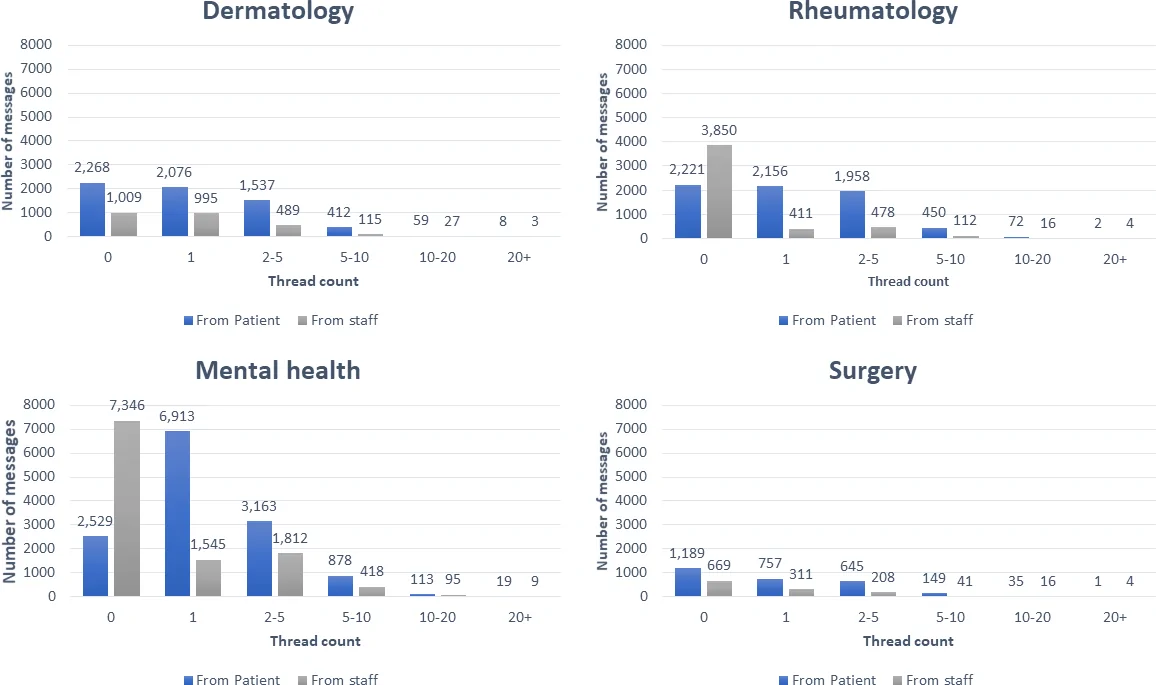
Number of replies within each conversation.

When messages were responded to, the majority of conversations initiated by patients had a single response. For example, across messages sent from patients, 51% (2076/4092) in dermatology, 46% (2156/6859) in rheumatology, 62% (6913/13,615) in mental health, and 48% (757/2776) in surgery had a single reply. Across messages initiated by clinical staff in dermatology (1009/2638, 61%) and surgery (311/1249, 54%), the majority of messages had a single response. In rheumatology (478/4,871, 10%) and mental health (1812/11,225, 16%), staff messages were more likely to receive 2 to 5 replies. Across all clinics, there were very few instances in which conversations had more than 5 replies. This suggests that messaging could be used mostly to communicate simple information or send inquiries that were easy to resolve and did not require a lot of clarifying follow-ups.

The majority of messages by patients and providers were primarily sent during regular working hours (8 AM to 5 PM). When messages were sent outside working hours, they were sent mostly by patients and not providers (Figure 6). Across provider messages, the majority were sent during regular working hours: 90% (32,403/36,022) in dermatology, 93% (25,826/27,739) in rheumatology, 95% (85,976/90,899) in mental health, and 97% (37,107/38,370) in surgery. While providers handled the majority of their messages during regular work hours, they still respond to substantial volumes after hours—3619 messages in dermatology, 1913 in rheumatology, 4923 in mental health, and 1263 in surgery over the evaluation period.

**Figure 6.**
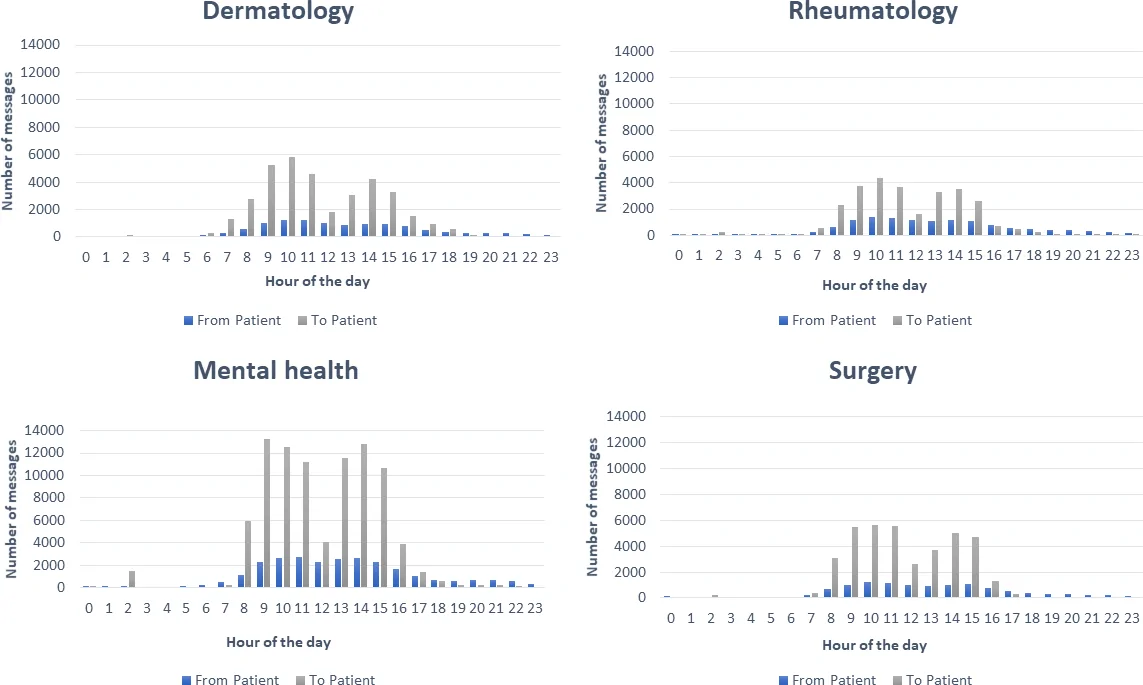
Time of day messages were received from and to patients.

### Qualitative Results

#### Qualitative Results Overview

We interviewed 19 participants across the four participating clinics, including 11 health care professionals (physicians), 5 administrative staff personnel (clinical flow supervisors, coordinators, and secretaries who triage messages), and 3 patients. Interviews were distributed across clinics as follows: 5 in rheumatology, 5 in surgery, 4 in mental health, and 2 in dermatology; 3 additional patients were interviewed independent of a specific clinic (Table 3). No nursing staff were interviewed despite recruitment efforts, and no participants dropped out of the study. Three main themes were identified: (1) physicians had an overall poor experience with messaging across most departments, (2) there was a high value proposition for the use of messaging among patients and administrative staff, and (3) there was poor engagement of all users in decisions around the use of messaging. Each theme is described below, supported by illustrative quotations.

**Table 3.** Qualitative interview participants by clinic and role.

Clinic	Total participants, N=19	Administrative staff, n=5	Physicians, n=11
Rheumatology	5	2 (1 involved in triage)	3
Surgery	5	2 (1 involved in triage)	3
Mental health	4	1	3
Dermatology	2	0	2
Subtotal (clinics)	16	5	11
Patients (not clinic-specific)	3	—^a^	—

a Not applicable.

#### Theme 1: Physicians Had an Overall Poor Experience With Messaging Across Most Departments

Physician’s challenges with messaging was particularly pronounced in departments that received a high volume of messages, with no additional staff support available to triage and respond, such as the rheumatology department. Many physicians indicated the notable anxiety they experienced when opening their inboxes due to the uncertainty of how many messages they must respond to, and about having to stay extra hours after clinic to respond to messages.

The amount of anxiety it gave physicians….when we go to click on that folder, we get a little … nervous cause we're like, OK, how many, how bad, how extensive? People write a lot. And it was really, really impacting, I think people’s wellness.

[Physician 4, rheumatology]

Several subthemes fueled this negative experience. First, messaging increased physicians’ workload. Both physicians and administrative staff acknowledged this.

I have to sort of adjust my clinics, extend my clinics, add clinics, et cetera, to try and accommodate some of these patients.”…..“I had to devote 30 to 45 minutes at the end of every day to deal with those messages, and I was getting increasingly angry about it because, it’s like, I’ve already had a full day. I’m already tired, and the last thing I wanna do is spend another 45 minutes answering these patients.

[Physician 1, rheumatology]

The workload varied across specialties and occasionally between clinics within a specialty. Clinics with higher patient volumes, higher patient-to-physician ratios, and those without triaging nursing support seemed to be more impacted but require further exploration. In the surgery department, nurses were assigned to physicians with whom they had a long-term working relationship, allowing them to learn the physician’s preferences and approaches to work more effectively together. Nurses in those clinics triaged and resolved most messages, and the surgery department usually could see patients quickly if messaging was not suitable. In other departments, although some triage was in place, integrated models of care around messaging were lacking, and most messages were still forwarded to physicians, even after being screened by a nurse or administrative staff member.

Second, there was a general feeling that patients were using messaging inadequately. Physicians reported that it was common for patients to message about new medical concerns, urgent medical or administrative requests, or even ask for advice on family members’ medical conditions or new medications prescribed by other physicians, which would be considered inappropriate context or urgency for use for ASM.

[Patients] will occasionally message thinking it’s relevant, or they can’t get a hold of another provider. If … it sounded completely unrelated like chest pain, [that’s outside of my scope, as] I’m [a] rheumatolog[ist]. [Patients have said] I have worse shortness of breath after COVID I can’t get a hold of my family doctor. We were the only accessible portal to health care that [patients] had because we were just always available [in] this portal, but … it’s not within my expertise … But now this is something urgent, chest pain that you … can’t ignore.

[Physician 2, rheumatology]

Most physicians were concerned that patient messages were often very long (several pages), and it took them a long time to review and respond to and likely better handled with synchronous visits.

Patients have access in a way that [they] didn’t in the past. All [patients] have to do is send [a message]…In the past, patient[s] wouldn’t have called and [asked for the same], they would have understood that if they have more questions, they really need to set up another appointment.

[Physician 11, surgery]

Physicians often felt that messaging could not offer the same quality of care as a synchronous visit (virtual or in-person), especially for new issues, resulting in tension between physicians and patients regarding how messaging is to be used. From the physician’s perspective, the messaging should be used for straightforward follow-up requests or queries related to their last clinical visit with that provider, rather than new and more urgent scenarios. Physicians noted that patients also use messaging to indicate frustrations about the care they received or challenges to change rather than follow-up medical care for the pertinent issue after their last appointment.

The third subtheme driving physicians’ negative experience was the lack of compensation for messaging workload and the increased liability it entails. Physicians were particularly concerned about being expected to use messaging to complete tasks that would typically be compensated. Some physicians said they refused to respond to clinical questions through messaging and always instructed patients to make appointments.

(We are) not compensated in any way, shape, or form. So, dealing with a problem, looking at pictures, doing whatever and receiving no compensation for that? And it’s just a giant uncompensated increase in workload, and I don't think it helps.

[Physician 11, dermatology]

Others mentioned that if there were a way to automate message responses, they would not have an issue with it, but they were not willing to spend effort on uncompensated tasks.

I think if it were totally automated, I think people would have no problem with it, but if it did require time and effort it should be compensated … I don’t think you’d have physicians who could participate.

[Physician 11, surgery]

Unfortunately, physicians’ ability to schedule patients varied across specialties. While some physicians (eg, surgeons) reported that they usually had the means to book a follow-up appointment to review the patient quickly, other specialties (eg, dermatology) indicated that they had limited capacity to offer an earlier appointment. This left physicians in precarious situations where, ethically, they felt obligated to provide care but could not see patients through other modalities. They were also unsure whether it was their responsibility to address medical concerns they became aware of through messaging that may be outside their scope of expertise. They also had varying perceptions of the guidelines regarding patient response times, and none of the interviewees had received training on using messaging in medical care. Physicians had to either stay after hours at the clinic or see patients on days when they were not expected to, just to accommodate these additional requests. Liability was also questioned regarding patients sending messages about urgent medical conditions like chest pain or suicidal ideation. Despite departmental rules allowing physicians several days to respond, physicians felt they needed to review messages more often, as there was always a risk that patients would send urgent medical requests and they would be held responsible. Physicians were also concerned about making medical decisions with incomplete information, which they sometimes had to do because they could not see patients in person.

#### Theme 2: There Was a High Value Proposition for the Use of Messaging Among Patients and Administrative Staff

The most clearly defined value proposition for using messages was that they were convenient for patients and improved access to their health care team, meeting their needs outside of scheduled appointment times with their respective clinician.

I think it makes it easier [for patients]. Like instead of phoning and maybe being put on hold to, you know, leave a message … [patients] can just send a quick message. It gets to us quicker.

[Physician 13, mental health]

Almost everybody expressed this sentiment, including physicians, patients, and administrative staff. Patients liked the ability to send messages at any time of day; this improved their access to care and was often preferred to the telephone.

I just want to add to that, the majority of my messages are after hours, so I think the patients like that option. So, when I come in first thing in the morning, boom, the first thing I check is my EPIC messages. And then I go to my voice conference stuff. So, I think it’s a good system.

[Physician 6, surgery]

The messaging system was also more efficient, allowing appointment changes and physician responses to be made more rapidly than by phone. Some patients also reported that it was easier to keep track of written communication because they could go back and review what was said, rather than trying to remember it during a visit.

From a patient perspective, you could be [in a situation where you want to say:] I can’t take an incoming call because I’m at work. I don’t have privacy [when] I’m on zoom meeting or I’m not awake because I’m nocturnal.

[Patient 28]

There were also several benefits to using the messaging system for administrative staff. Administrative staff reported that messaging was faster to manage than voicemail (as voice messages must be transcribed and entered into the chart). Messaging also saved time, was private, and more accurate because it did not require matching and transcribing a voice message to a specific patient, since it was already linked in the system to a patient.

…you have to transcribe the voicemail, and you have to do that by hand copying. So, then it’s an hour or two in the morning, just transcribing voicemail. Then you’re looking up in EPIC all the information; then you’re making a phone call back to the patient; whether they’re there or their voicemail; if it’s voicemail, or they don’t have voicemail, then you’re calling the patient back; or the patient didn’t understand, so the patient is calling back. So, it turns into a much longer process than just looking it up and typing it back.

[Administrative staff 3, rheumatology]

The messaging system was viewed by administrative staff and patients as providing better access to patient care, especially for structurally marginalized patient populations. Examples given were improving access for patients who have to work during the day and have limited access to work breaks to be able to accommodate synchronous virtual or in-person appointments, better access for patients with hearing impairments, who may find it difficult to communicate over the phone, and improving accessibility for out-of-town patients who have to pay long-distance phone charges to call the clinic.

Patients who are already at a disadvantage, patients who are working in an industry where they can’t sit in an office and take a private phone call anytime they like, disabled patients, low income patients, patients out of town, all of the most vulnerable patients were more disproportionately affected than a patient who has an office or is retired or has free time and can sit on the phone and wait for half an hour for somebody to pick up, or can call 10 times in an hour to finally get through to a live person, or who can leave a voicemail and wait, possibly two days for something they felt was urgent.

[Administrative staff 3, rheumatology]

#### Theme 3: Poor Engagement of All Users in Decisions Around the Use of Messaging

General tension existed in some departments about staff not being consulted in the hospital’s decision to turn on messaging and also shut down the direct patient-to-physician messaging feature in some clinical areas. Given the high value proposition described above, administrative staff reported they were not involved in making decisions about the use of messages, how they were implemented, or workflows. According to administrative staff, some patients also complained that messages were turned off. Physicians, on the other hand, were not initially involved in the decision to implement patient-to-provider secure messaging. They also felt they were not adequately engaged in the implementation process, provided training, or in developing adequate workflows to support it. At the hospital, patients could message physicians for up to two years after their visit, but patients could be discharged from care after a single visit. Keeping the messaging system open provided patients with access they would not have had previously, and physicians felt they should have been consulted when the hospital made decisions surrounding the design, functionality, and implementation of secure messaging. This window was eventually reduced to 5 days.

I felt it was an equity issue that we turned it off, and I felt like we could have or should have proposed other solutions before just blanket turning it on or off. Maybe we should have done a needs assessment. Maybe we should have looked at who this is affecting. Maybe we could have found better ways to make the messages more succinct or more pointed or more directed. So that the physicians didn’t feel the weight of what they thought was an inordinate amount of work.

[Administrative staff 3, rheumatology]

### Mixed Methods Results

The areas that we identified as converging related to data on message volume, message timing, and the reported roles of various users within each clinic in message management. Table 4 presents a joint display summarizing the 3 areas of integration.

**Table 4.** Joint display: integration of quantitative and qualitative findings.

Domain	Quantitative finding	Qualitative finding	Integration type	Interpretation
Role of dedicated triage staff	Physicians averaged 12.6 (SD 7.6) messages/month (rheumatology) and 7.6 (SD 4.3) messages/month (mental health) in clinics without nursing support. Nurses handled 8‐27 messages/month in dermatology and surgery, leaving physicians with fewer messages to respond to.	Physicians in rheumatology and mental health described high workload and anxiety managing inboxes without support. Physicians in surgery described nurses resolving most routine questions before escalation.	Convergence	Nursing triage capacity, not message volume alone, differentiates physician burden across clinics.
Patient-to-physician ratio	Dermatology and rheumatology had higher patient-to-physician ratios (71:1 and 86:1) than mental health and surgery (42:1 and 49:1).	A dermatologist raised high patient load as a contributing factor to workload burden in interviews.	Convergence	Ratio is a contextual factor but not sufficient alone to explain outcomes (see Discussion: surgery vs. mental health contrast).
After-hours workload	Only 2%‐10% of provider messages were sent or responded to outside regular hours across clinics.	Physicians, particularly in rheumatology, described significant distress and time spent responding to messages after hours.	Divergence	Low proportions still represented thousands of absolute after-hours messages; perceived burden may reflect unpredictability and cognitive load rather than time spent responding alone.

#### The Role of Dedicated Staff in Handling Secure Messages

Overall, physician experiences with messaging were more negative in rheumatology and mental health, and based on the quantitative data, these were departments with higher message volumes. In rheumatology, an average of 12.6 (SD 8.0) messages per physician were sent per month, while in mental health, the average number was 7.6 (SD 4.4) during the study period. In contrast, in dermatology and surgery, physicians had, on average, less than one message per month to respond to (mean 0.4, SD 0.4 in surgery and mean 0.2, SD 0.2 in dermatology), likely because most were handled by nurses. Nurses responded to 19 and 27 messages per month in the dermatology and surgery departments, respectively. Despite this, physicians in the dermatology department indicated that they felt many messages were forwarded directly to them without much effort from the nurses to resolve the clinical question. In the surgery department, nurses would handle most common postoperative questions, but anything outside routine care was handled by the physician. The rheumatology and mental health departments, on the other hand, did not have regular nursing staff participating in messages.

…in other models you would have some support; you don’t have that support here. And, so unless we have additional nursing support or physician assistant support or something along those lines, the messages… I don't think are going to work. We need additional help.

[Physician 4, rheumatology]

This means that having staff dedicated to triaging and responding to messaging significantly improved the physician experience, as only messages that required escalation with the physician’s input reached their inbox. Unfortunately, in the hospital’s current models of care, messaging was not properly embedded within local workflows to triage based on request, and physicians received many inappropriate messages, ultimately resulting in a negative experience with messaging as described above. It is important to note that at the hospital, while nurses were responding to messages, there were no dedicated positions of nursing staff that were tasked mainly with handling messages. The question remains as to what extent responding to messaging increased the nurses’ workload burden, as we were unable to recruit nursing staff across all departments.

#### High Patient-to-Physician Ratios

Another factor affecting the perceived workload burden from messages was the patient-to-physician ratios, and these naturally varied quite a bit across specialties. Different specialties require different amounts of time per patient, which results in variations of patient-to-provider ratios across specialties. Some specialties, such as dermatology, are more procedure-focused and may have faster turnaround versus specialties like mental health, where patients may participate in longer-term treatment programs. A dermatologist pointed out in the interviews that their patient-to-physician ratios were high and our records confirmed that dermatology, alongside rheumatology, had higher patient-to-physician ratios (71:1 and 86:1, respectively), while mental health and surgery had lower patient-to-physician ratios (42:1 and 49:1, respectively). It is likely that this larger number of patients has the potential to generate larger volumes of messages. Combining this with a scenario where physicians also have no dedicated staff to respond to messages (such as in rheumatology), the demand on physicians becomes too high, leading to the negative experiences reported in departments such as rheumatology.

#### Working After Hours

Physicians had significant concerns about the workload resulting from messages that translated into working after hours, and yet we found that the messages sent after hours represented only a small fraction of all messages. The EHR data showed that dermatology and rheumatology had the highest percentage of messages responded to outside regular hours, but it was still only at 10% (3619/36,022) in dermatology and 7% (1913/27,739). Rheumatology was the department in which physicians spoke a lot about working after regular clinic hours. The mental health department responded to 5% (4923/90,899) of messages outside regular hours. Surgery had smaller proportions of messages being responded to after hours: 3% (1263/38,370). As mentioned earlier, despite these percentages seeming low, this translated into thousands of messages responded to after hours over the study period, so the physicians were still affected significantly.

Now they just send me a message directly with whatever … And then I have to assess this…, I’d have to look up their record, I have to read their last follow-up note, I have to go on to Connecting Ontario to look at their blood work. And so basically, it’s like I’m doing a medical assessment at the end of the day when they don’t have an appointment, but I still have to assess this because they sent me a message…… It increases my workload.

[Physician 1, rheumatology]

## Discussion

### Principal Findings

Our study showed a steady increase in message volume at the onset of the pandemic, which continued to rise in 2022 in departments where the Medical Advice Messaging pathway remained open (mental health and surgery). Even though the message threads were short, there was a significant increase in workload, and many messages were responded to outside regular clinic hours. Aside from the surgery department, there was a generally negative physician experience with messaging. Factors contributing to this negative experience included the perception that patients were sending inappropriate messages due to inadequate triage, an increased workload, and a lack of compensation amid increased liability. Administrative staff and patients appreciated the use of messages; however, low engagement with decisions around the use of secure messaging led to a generally negative experience.

The higher proportion of patient messages sent in the evenings indicates that patients are using secure messaging as a convenient way to communicate with their care teams outside traditional office hours, rather than leaving voicemail messages. This is consistent with literature showing that patients value the flexibility of asynchronous communication [6,27]. However, this pattern also highlights an implementation challenge: without clear boundaries around expectations, message response times, health care professionals may feel compelled to check and respond to messages during evening hours, contributing to the workload concerns and burnout risk described in our qualitative findings. This underscores the importance of establishing explicit expectations about response timeframes and protecting after-hours time as part of secure messaging implementation strategies. There have been many reports of increased message volumes across the health care system before and during the pandemic [12,28,29]. This increased demand drives clinicians to respond to messages outside clinical hours, as reported here and elsewhere. For example, Akbar et al [30] reported that 37% of the time physicians spent responding to messages in primary care occurred outside regular work hours, and physicians in their study spent about an hour a day responding to messages. They also reported that physicians’ time spent in the inbox was mostly spent before or after clinic hours or during lunch, suggesting that messaging was not adequately integrated into their daily clinic routine. Martinez et al [12] showed that for every 10 additional messages sent by clinical staff, 12 more minutes were spent working in the EHR outside regular clinical hours. This need to respond to messages outside clinical hours is likely due to the general increased workload associated with messaging. Holmgren et al [29] showed that each patient message increased in basket time per day by 2.32 minutes, so it is easy to see how these larger volumes of messages can easily add up, and clinicians may struggle to respond to all of them in between clinical visits.

Among the 4 clinics we examined, only surgery offered a better experience for physicians. The best model of messaging use was at clinics where staff were assigned to triage messages and a system was in place to allow a substantial proportion of messages to be handled by the triage staff, with adequate workflows to escalate messages when necessary to the most responsible physician. Unfortunately, in most clinics, the patient-to-provider messaging was not adequately integrated into workflows with training and adequate change management support. Messaging often introduced situations where patients had to be seen for an in-person or virtual appointment, but no such appointments were available for months ahead in some specialty practices. This created additional capacity challenges for already overwhelmed physicians. A proper integration of messaging requires a proper input, throughput, and output system [30] in place that can handle all patients, irrespective of whether messaging alone or messaging in combination with other appointment types is necessary to manage patient care. Ultimately, there is a need for proper “channel management” of visits [31]: the process of recommending to patients the appropriate “channel” or visit modality (messaging, phone, video, and in-person) based on patient, problem, and clinician characteristics. Unfortunately, our study shows that clinics often lack the ability to offer multiple channels or switch between them, and both patients and providers lack guidance on selecting the best channel.

This increased administrative burden on physicians naturally translated into a poor experience with messaging. Despite physicians’ negative experiences, all participants, including clinical and administrative staff, recognized the high value of messaging for patients, consistent with prior evidence [6,16].

Many of the individual factors contributing to physicians’ negative experiences, workload [19,30], lack of compensation [6], lack of clarity around liability issues [20,31], and inappropriate or insufficient information in messages [15,16], have been documented previously. Our contribution is in showing how these factors combine differently across clinical contexts to produce divergent outcomes. Notably, surgery and mental health had similar patient-to-physician ratios (49:1 and 42:1, respectively), yet reported markedly different experiences. This suggests that ratio alone does not determine outcomes; rather, the presence of dedicated nursing triage and the nature of the clinical content appear more decisive. Surgical messages were largely protocol-driven, acute, postoperative questions well-suited to delegation to nurses with established physician relationships. Mental health and rheumatology messages more often involved chronic, longitudinal, or clinically nuanced concerns requiring physician, or therapist, level judgment, with no triage infrastructure to absorb this volume. This distinction may explain why triage support alone was not sufficient. The type of clinical content also determines whether triage can meaningfully offload physician work.

This distinction also helps explain the low uptake of Ontario 2024 billing pilot to date. Our findings suggest that physicians’ negative experiences with messaging were driven less by the absence of compensation alone than by the combination of uncompensated work, absent workflow integration, and limited ability to fully resolve issues through asynchronous communication. A billing code addresses only one of these three barriers. Without parallel investment in triage staffing and workflow design, and without engaging physicians in the design of messaging models, compensation alone may be insufficient to shift adoption, which may account for the pilot’s limited uptake so far.

So how does one integrate messages into pre-existing workflows? Our study highlights several important insights. First, the successful integration of messaging requires thoughtful integration into existing workflows and possibly the creation of new clinical pathways that allow patients who cannot be resolved through messaging alone to move through the system through other visit modalities. Second, proper strategies for patient expectations, engagement, and training on the use of messaging should be in place that explain to patients how to use messaging, which use cases are most appropriate, and properly set their expectations [15]. Hefner et al [18] recently conducted a study that engaged patients and providers in developing strategies for successful training programs at the patient, portal, physician, and health system levels to improve patient-provider secure messaging. Organizations can use these strategies to prepare such training programs for both patients and staff, and, when implemented, can reduce the number of inappropriate messages and improve physicians’ experiences with patient-to-provider messaging. Providing physicians with compensation can also be a step in the right direction for the proper integration of messaging into health care systems. In Ontario, Canada, the government is currently running a pilot exploring the impact of paying physicians who participate in the pilot for secure message encounters, with the aim of examining the possibility of adding it to the suite of physician services covered by OHIP [32]. So far, interest in the pilot has been low, which may further support our hypothesis that secure messaging is poorly integrated into current models. Finally, our study revealed significant variation in how messages were used across specialties and who was available to respond. Adequate support staff to triage different types of messages, and escalating to the most responsible physician only when appropriate, can ensure “low-value” messages do not increase the burden on care teams. Future studies should focus on exploring these variations across specialties. Future studies can also explore AI draft messaging to reduce physician burden, workflow, or triaging, while supporting patient care for efficient messaging to meet care needs and access [33].

While this study used an inductive, exploratory approach given the limited prior research in this setting, our findings can be usefully interpreted through the nonadoption, abandonment, scale-up, spread, and sustainability (NASSS) framework for technology adoption in health care [34]. Barriers at the organizational level (absent triage workflows and lack of role clarity) and the wider system level (absence of remuneration and exclusion of physicians from implementation decisions) appear to have been more consequential to adoption than characteristics of the technology itself. This suggests that efforts to scale ASM, including Ontario 2024 billing pilot, are likely to face continued nonadoption unless organizational and system-level barriers are addressed alongside financial incentives.

### Limitations

There are several limitations associated with this study. First, the findings are limited to a single urban ambulatory academic site in Ontario, limiting the generalization of the findings to other settings, particularly rural or community hospitals, or other sites outside of Canada, with different payment models or health system structures. In addition, the findings are limited to only four ambulatory-care hospital-based specialties, and the number of clinicians interviewed was relatively low, which may not sufficiently represent the diversity of experiences across all user types. Further, few nursing staff and patients were included in the evaluation, despite our efforts to do so. As our evaluation indicates, nurses play a crucial role in providing clinical care through messaging in successful models of care, but their perspectives need to be examined more thoroughly in clinics where they handle the majority of messages and merits further exploration in a future study. The low patient participation may reflect the relatively low adoption of messaging at the hospital during the study period, which may have resulted in a small pool of eligible participants.

We used convenience and snowball sampling, which come with limitations despite being widely used in qualitative research. Finally, we relied solely on EHR-based quantitative data, which has its limitations. This limited assessment of messaging content constrains our ability to assess message complexity, appropriateness, and the message’s urgency. In addition, some messages may have been handled through alternative workflows (eg, in-person follow-ups or phone calls to clinics) that were not captured. We calculated the number of unique patients and providers based on having at least one appointment per month within their respective clinic, rather than message use, which would have deflated message rates per provider and patient. In addition, other EHR-based limitations include the fact that messages were grouped by recipients rather than by responders. Some forwarded messages may have been misclassified. Administrative users were not clearly defined within the EHR system, making it difficult to determine how many messages they managed. Many administrative staff members opened messages sent to providers, potentially leading to misclassification of who handled them. While we reviewed process-levels of messaging (ie, volume and workload), it would be important in future studies to assess content and quality (patient, outcomes, safety incidents, or clinical decision-making ability). Finally, as we conducted a convergent mixed methods study, the qualitative and quantitative data were collected during the same period and merged after data collection was complete in both streams, which limited our ability to inquire about trends observed in the quantitative data.

### Conclusion

In conclusion, the adoption of digital technologies during the pandemic has provided patients access to providers through channels like messaging, but yet these are not fully integrated into the care model. Messaging could be a fully integrated visit modality, but it needs to be supported by appropriate triage workflows and channel management. Asynchronous messaging can reduce unnecessary interruptions, improve access, distribute workload across team members, and increase efficiency, which can be valuable in ambulatory care and warrants further exploration. Better integrated care models that effectively manage patients across different visit modalities, such as in-person, telephone, video, and messaging, are likely to lessen the burden on physicians while also enhancing the experience for patients and administrative staff.

## Abbreviations

APQIP: Assessment Process for Quality Improvement Projects
ASM: asynchronous secure messages
COREQ: Consolidated Criteria for Reporting Qualitative Research
EHR: electronic health record
NASSS: nonadoption, abandonment, scale-up, spread, and sustainability
OHIP: Ontario Health Insurance Plan
WCH: Women’s College Hospital
